# Participant experiences of genome sequencing for rare diseases in the 100,000 Genomes Project: a mixed methods study

**DOI:** 10.1038/s41431-022-01065-2

**Published:** 2022-03-09

**Authors:** Michelle Peter, Jennifer Hammond, Saskia C. Sanderson, Jana Gurasashvili, Amy Hunter, Beverly Searle, Christine Patch, Lyn S. Chitty, Melissa Hill, Celine Lewis

**Affiliations:** 1 NHS North Thames Genomic Laboratory Hub, Great Ormond Street Hospital for Children NHS Foundation Trust, London, UK; 2 Genetics and Genomic Medicine, UCL Great Ormond Street Institute of Child Health, London, UK; 3 Our Future Health, London, UK; 4 Genetic Alliance UK, London, UK; 5 Unique – Rare Chromosome Disorder Support Group, Oxted, UK; 6 Genomics England, Queen Mary University of London, Dawson Hall, London, UK; 7 Society and Ethics Research, Wellcome Genome Campus, Cambridge, UK; 8 Population, Policy and Practice Department, UCL Great Ormond Street Institute of Child Health, London, UK

**Keywords:** Social sciences, Genomics

## Abstract

In this mixed methods study, a survey and in-depth interviews were used to explore whether decision regret and the psychological impact of receiving genome sequencing (GS) results differed between parents and patients, and between those who received a genetic diagnosis and those who did not. Participants (*n* = 77) completed a survey that included the Decisional Regret Scale (DRS) and an adaptation of the Multidimensional Impact of Cancer Risk Assessment (MICRA) at least 12 months after consenting for GS for rare disease diagnosis in the 100,000 Genomes Project. Survey participants were invited to take part in an interview and 39 agreed; 12 with a diagnosis, 5 with variants of uncertain significance, and 19 with no pathogenic findings identified. Both survey and interview findings indicated that decision regret was low. DRS scores revealed no differences in levels of regret between parents and patients, or between those with a diagnosis and those without. Though MICRA scores indicated minimal evidence of negative psychological impacts of receiving GS results, subscale analysis revealed greater distress and uncertainty for parents compared to patients. Receiving a diagnosis was found not to influence MICRA scores, supporting interview findings of both positive and negative emotional and psychological impacts irrespective of a genetic diagnosis. Our findings have implications for policy and practice as GS is integrated into the UK and worldwide; notably, that expectation-setting is critical when offering GS, and that post-test counselling is important regardless of the GS result received, with parents perhaps needing additional emotional support.

## Introduction

Genomic tests such as genome sequencing (GS) and exome sequencing (ES) are increasingly being used in clinical settings to aid the diagnosis of rare and inherited diseases in children and adults. The potential benefits of genomic testing include increased knowledge, modifications to medications, procedures or treatment options, improved long-term clinical management, a clearer prognosis, information about recurrence risk and risks for other family members, as well as opportunities to obtain tailored information and support [1–3]. Not all individuals having GS will receive a diagnosis and some findings will reveal a variant of uncertain significance (VUS). However, it is anticipated that diagnostic yields from ES and GS will continue to increase as our knowledge grows [4]. The 100,000 Genomes Project (100kGP) was a hybrid clinical and research project designed to prepare for the implementation of GS in the National Health Service (NHS). Patients with cancer or selected rare and inherited diseases, and parents and relatives of these patients were enrolled in the 100kGP between 2015 and 2018 and consented to receive main findings from GS, to contribute their data for research, and had the option to receive clinically actionable ‘additional health-related findings’ [5]. Most of the research around participant experiences of the 100kGP has focused on motivations for uptake and the consent processes [6–9]. However, to inform both policy and clinical genetics practice as GS is introduced into routine care in the UK, we need insight into participants’ experiences of receiving GS results.

Several studies have reported limited psychological harms for adult patients and parents following the receipt of genomic test results [10–15]. For example, a recent exploratory meta-analysis of psychological outcomes related to result disclosure at seven sites offering ES/GS found that, in general, these results did not lead to negative psychological effects, and that there were no observable differences in psychological outcomes between participants with and without a diagnosis [10]. The authors did, however, note while negative emotions and distress were generally infrequent, there was variability with somewhat greater distress at the sites that included paediatric populations. In addition, several studies have found that there can be negative psychological outcomes, particularly for parents of children offered ES or GS for rare disease diagnosis [16–19]. For those parents that receive a diagnostic result, this can result in relief but also worry, fear, loss of hope, frustration at a lack of information and disappointment if management is not altered [16–19]. Parents who do not receive a diagnostic result have also been found to experience negative psychological outcomes including frustration and disappointment at the remaining uncertainty [16, 19]. In addition, decision regret (DR) has been observed in parent cohorts. In a survey study by Wynn et al. [17], around 20% of parents expressed some regret over the decision to have testing (irrespective of the test result), and parents who interpreted their child as receiving a diagnostic result, experienced more negative psychological outcomes than those parents who did not, highlighting the complexity of psychological outcomes for this particular cohort.

If there are differences across particular groups (e.g., parents vs. patients or those with a diagnostic result vs. those without a diagnostic result), then this will help professionals to better understand the perspectives of adult patients and parents and have important implications for counselling and support following result disclosure. To build on the prior research, and to examine explicitly the experience of participants taking part in the 100kGP who include both parents and adult patients, we conducted a mixed methods study involving surveys and interviews at two timepoints: time of testing (T1) and 12–18 months later (T2). In this manuscript, we report on DR and the psychological impact of GS results identified from the T2 survey and interviews. Our research questions were: (1) Does DR and the psychological impact of receiving a GS result differ between parents and patients? (2) Does DR and the psychological impact of receiving GS results differ between those who received a genetic diagnosis and those who did not.

## Methods

### Mixed methods design

Our overarching mixed methods study design [20] comprised cross-sectional surveys distributed to 100kGP participants at two timepoints (time of testing (T1) and 12–18 months later (T2)) followed by in-depth interviews with a subset of survey respondents. Working within a pragmatist paradigm (a worldview that focuses on “what works” rather than what might be considered absolutely and objectively “true” or “real” [21]), we drew on both qualitative and quantitative assumptions to investigate different facets of the same phenomena (‘complementarity’). Quantitative data collection and analysis preceded qualitative data collection and analysis, and both the quantitative and qualitative components were given equal status. Data were analysed separately and integrated at the final stage in order to answer the research questions (composite analysis) [22].

### Development of the T2 survey and interview topic guides

Surveys and interview topic guides were developed by SCS and CL, with input from the wider study team and members of the advisory group (see Supplementary materials). The survey was designed to assess attitudes, knowledge, decision making and the impact of receiving GS results. Survey findings reported here are DR, measured using the Decisional Regret Scale (DRS) [23], and the psychological impact of receiving GS results, measured using an adapted version of the Multidimensional Impact of Cancer Risk Assessment (MICRA) [24]. We used 17 items: 1–12; 14, 15 (addition of the word, “negatively”); 16; 21 and added an item (13; “Being uncertain about what the result means for the management of my relative’s rare condition”.).

The interview topic guide included: motivations for participating in the 100kGP, decision making, communication of results, experience of receiving GS results, and understanding and impact of GS results. Interview findings reported here relate to DR and impact of GS results.

### Participants and recruitment

Participants were recruited from six London hospitals that were part of two Genomic Medicine Centres involved in recruiting probands and their relatives into the 100kGP. Participants included: (a) adult patients with a genetically undiagnosed rare disease, (b) parents of children with a genetically undiagnosed rare disease and, (c) relatives of patients with a genetically undiagnosed rare disease undergoing GS to help identify the gene variant causing the proband’s condition. Dyads from a single family were eligible. Approximately 12 months after completing the T1 survey (conducted between 1 July 2017 and 30 September 2018), respondents (*n* = 504) were invited to complete either a paper or online version of the T2 survey via SurveyMonkey. Respondents who reported receiving a GS result, which could be a diagnostic result, a VUS result or a no primary finding result, were invited to take part in an interview.

### Data analysis

#### Quantitative surveys

Only survey data from respondents who reported receiving a GS result (*n* = 77) were analysed. All analyses were conducted using R 4.0.2 [25]. To answer our research questions, we investigated the relationship between participant type (parent/patient) and diagnosis status (genetic diagnosis/no genetic diagnosis) on our outcome variables: DR and psychological impact. We considered those with a VUS result as having received no genetic diagnosis. Since the residuals of the DRS scores deviated from normality even after transformation, scores were classified into three categories that have been used elsewhere in the literature [26] where 0 = no regret; 5–25 = mild regret; and ≥30 = moderate to high regret. *χ*^2^ tests of independence were used to assess associations between regret and our two variables. For MICRA scores (for which the residuals were approximately normally distributed), we performed multiple regression with bootstrapped simulations (*R* = 1000) in which 95% confidence intervals and *p* values for the model estimates were obtained. As neither DRS nor MICRA scores were the primary outcomes for the survey, a priori power calculations to find subgroup differences were not performed.

#### Qualitative interviews

Interviews were conducted by four researchers (CL, JH, MP, MH) and transcribed verbatim. Data were analysed following the principles of codebook thematic analysis [27]. An initial draft codebook, developed by JH and CL, was informed by the aims of the study (deductive component). These two researchers then read and independently coded three transcripts, adding codes not covered by the original codebook (inductive component). Additional codes were discussed and the resulting revised codebook was used to code the remaining transcripts. NVivo 12 (QSR International, Australia) was used to group codes into thematic categories which were then refined during discussions between JH, MP, CL, and MH. To facilitate comparisons between participant sub-groups (i.e., parents and patients/participants with and without a diagnosis), illustrative quotes from each participant were charted in a matrix against themes relevant to the research questions.

### Quantitative measures

#### Decisional Regret Scale (DRS)

Participants provide ratings on a 5-point Likert scale (1 = strongly agree, 5 = strongly disagree) for five items regarding their DR. Items two and four are reverse coded so that, for each item, a higher number indicates greater regret. Scores are converted to a 0–100 scale and can range from 0 (no regret) to 100 (high regret). Cronbach’s alpha revealed good internal consistency (*α* = 0.82).

#### Multidimensional Impact of Cancer Risk Assessment (MICRA)

Participants provide ratings on a 4-point scale (Never = 0, Rarely = 1, Sometimes = 3, Often = 5). Seventeen items were taken from the original 25-item MICRA and comprise three subscales: Distress, Uncertainty, and Positive Experiences. Scores for this adapted version could range from 0 (no impact) to 85 (high impact). Cronbach’s alpha revealed good internal consistency across the whole scale (*α* = 0.82) and for each subscale (Distress: *α* = 0.85; Uncertainty: *α* = 0.88; Positive experiences: *α* = 0.87).

## Results

### Participant characteristics

Of the 296 unique T2 surveys received (58.7% response rate), 77 participants (26%) reported receiving a GS result (see Supplementary materials for participant characteristics), and were invited to interviews: 39 (50.6%) agreed (27 did not respond, 10 declined, and one could not be reached by phone). One participant was excluded as their results were not received through the 100kGP. Interview participants included two parent couples. Interviews lasted between 17:50 and 61:01 min (median = 31:06 min); 27 were conducted by telephone and 11 by video call.

## Survey results

### Decision regret

Of the 73 participants who provided data for this assessment, 32 were patients and 41 were parents. However, 16 parents comprised eight dyads. To avoid associations between paired data, DRS scores from one parent in each dyad was excluded through random selection. Across all participants (*n* = 65), the mean DRS score was 10.31 (SD = 12.12, range = 0–50) and the median score was 5 (IQ1 = 0, IQ3 = 20) which, given the total maximum possible score of 100, shows that DR was low (Table 1). Viewing the data in terms of the discrete categories corroborated this finding and showed that few people (*n* = 6; 9%) had high levels of regret (see Supplementary materials). In answer to our research questions, *χ*^2^ tests revealed no association between DR and participant type [*χ*^2^ (2, *n* = 65) = 2.03, *p* = 0.36] or between DR and diagnosis status [*χ*^2^ (2, *n* = 65) = 1.23, *p* = 0.54] indicating that regret did not differ between parents and patients or between those with a diagnosis and those without.

**Table 1:** DRS scores split by participant type and diagnosis status.

	Participant type		Diagnosis status	
	Parent	Patient	Genetic diagnosis	No genetic diagnosis
	*n* = 33	*n* = 32	*n* = 21	*n* = 44
Mean	9.1	11.6	9.0	10.9
95% CI	4.76	3.84	5.92	3.58
SD	13.43	10.66	13.00	11.78
Range	0–50	0–30	0–50	0–40
Q1	0	0	0	0
Median	5	10	0	7.5
Q3	15	20	15	20

*CI* confidence interval, *SD* standard deviation, *Q1* first quartile, *Q3* third quartile.

### Impact of receiving results from GS

Of the 71 participants who provided data, 31 were patients and 40 were parents. Fourteen of these parents comprised seven dyads so, in the same way as above, MICRA scores from one parent in each dyad were excluded. Across all participants (*n* = 64), the mean score (collapsed across subscales) was 17.22 (SD = 14.71, range = 0–63) and the median was 14 (IQ1 = 5.75, IQ3 = 26) which, given the maximum possible score of 85, indicates low negative psychological impact. For each of the following subscales, a multiple regression was performed with subscale score as the outcome variable and participant type and diagnosis status as the independent variables (see Table 2 for descriptive statistics).

**Table 2:** Descriptive statistics for MICRA scores split by subscale, participant type and diagnosis status.

	Participant type		Diagnosis status	
	Parent	Patient	Genetic diagnosis	No genetic diagnosis
	*n* = 31	*n* = 33	*n* = 21	*n* = 43
*Distress (possible scores range from 0 to 35)*				
Mean	7.8	2.2	5.0	5.1
95% CI	2.57	1.41	2.36	2.18
SD	7.25	3.85	5.19	7.08
Range	0–26	0–15	0–16	0–26
Q1	1	0	0	0
Median	7	0	3	1
Q3	12	2.5	7	9
*Uncertainty (possible scores range from 0 to 40)*				
Mean	11.6	6.4	9.0	9.1
95% CI	3.56	2.53	3.68	2.93
SD	10.05	6.91	8.09	9.51
Range	0–38	0–24	0–31	0–38
Q1	4	1.5	4	2
Median	9	4	7	5
Q3	17	9	12	16
*Positive experiences (possible scores range from 0 to 10)*				
Mean	3.0	3.1	4.2	2.5
95% CI	1.25	1.32	1.25	1
SD	3.52	3.60	3.52	3.25
Range	0–10	0–10	0–10	0–10
Q1	0	0	0	0
Median	2	2	5	1
Q3	6	6	6	3.5

*CI* confidence interval, *SD* standard deviation, *Q1* first quartile, *Q3* third quartile.

#### Distress

Results showed that parents reported higher levels of distress than patients (*β* = 2.87, [1.4, 4.32], SE = 0.74, *p* < 0.001) but that distress did not differ significantly for those with a diagnosis compared to those without (*β* = −0.31 [−1.68, 1.08], SE = 0.70, *p* = 0.662).

#### Uncertainty

Analysis revealed differences by participant type: uncertainty was higher for parents than patients (*β* = 2.65, [0.52, 4.72], SE = 1.07, *p* = 0.013). Uncertainty did not differ, however, between those with and without a diagnosis (*β* = −0.27 [−2.53, 2.09], SE = 1.18, *p* = 0.821).

#### Positive experiences

Results indicated that positive experiences did not differ between parents and patients (*β* = −0.13, [−1.0, 0.70], SE = 0.43, *p* = 0.770), nor between those with a diagnosis and those without (*β* = 0.85 [−0.12, 1.82], SE = 0.50, *p* = 0.087).

## Interview results

### Decision regret? I would do it again tomorrow

All participants were emphatic that they had no regrets about their decision to have GS, regardless of the result they received or whether they were parents or patients (Table 3: Q1 and Q2). For parents and patients who had undergone multiple investigations prior to the 100kGP or who had been living with their condition for years, having GS was simply another testing option to get answers to long-standing questions about their health. For many participants, lack of regret was tied to being grateful for the opportunity to take part in the 100kGP with altruistic reasons playing a key role. Participants reported finding it rewarding to take part in something that could help others and benefit medical research. While no participants stated that they regretted taking part in the 100kGP or having GS, some were ambivalent. For example, one participant commented; “I wanted to take part mainly because of my interest in these things. But it wouldn’t have made any difference to me if I hadn’t” (P1—Patient—no genetic cause found).

**Table 3:** Participants’ experiences of receiving a GS result from the 100,000 Genomes Project.

	Quotation from interview
Q1	*“Oh I would do it again tomorrow… it is the best thing we could do for [child’s name] really, for us.”* P5—Parent—genetic cause found
Q2	*“Oh no, it hasn’t helped me at all, but I’m very happy to participate and I don’t regret participating in it.”* P42—Patient—no genetic cause found
Q3	*“I definitely wouldn’t have been threatened with him being removed from me, because there’s no way can they do that when it’s due to his underlying condition or his conditions…So knowing something like that would have been fantastic.”* P34—Parent—no genetic cause found
Q4	*“We get a couple of different grants for him to get specialist equipment for him. Nappies, because he’s still incontinent, and stuff like that for him. So it does really, really help now we’ve got the diagnosis.”* P18—Parent—genetic cause found
Q5	*“To be honest, I’ve never even thought about it, just because I see myself as sort of a fit and healthy twenty-three year old. I don’t really sort of think of myself as somebody with this like big condition. It doesn’t affect me massively, practically it hasn’t stopped me from doing anything.”* P13—Patient—no genetic cause found
Q6	*“If the boys start feeling [unwell] in any way or get the same symptoms, they can actually say to their GP or consultant or whoever “Oh by the way, my dad had this. We’ve got this you know “family [history]”.”* P3—Patient—genetic cause found
Q7	*“We’ve now been moved to, like, the specialist cystic kidney disease clinic, which I guess is for us a good thing because in the future I think if there’s any, like, research trials that are being done.”* P24—Parent—VUS result
Q8	*“To be honest, we have regular paediatrician appointments with him anyway, because he’s got other little health problems. So we go to cardiology, neurology and stuff like that. But as to his actual like condition, no, everything else carried on to how it was before.”* P18—Parent—genetic cause found
Q9	*“So yeah, it didn’t really, mean anything, because I already knew I had it, so it was just re-confirming to be fair.”* P4—Patient—genetic cause found
Q10	*“It’s a ‘we weren’t to blame’ which is a huge thing! Because you know, was it my age? Is it because I’m overweight? Should I have looked after myself better? Should I have picked up that there was things wrong during the pregnancy?…I felt guilt – it was like horrendous, actually. So that lifted.”* P22—Parent—genetic cause found
Q11	*“Well, relief that it wasn’t – I don’t quite know what I would have done if it had been family because my brothers being so much older, they obviously don’t need any kind of treatment, with having enough with different other conditions”*. P1—Patient—no genetic cause found
Q12	*“I just wanted to cry, it was awful you know? Because there was a finality to it. It’s almost like, well, that’s it…. I don’t know, but it was absolutely a feeling of grief.”* P21—Parent—no genetic cause found
Q13	*“It was a mixed feeling. I was feeling relieved because I guess in this situation the knowledge can really help you and it did actually. At the same time I was sad, yeah, simply sad, as you can imagine because it does give certainty of something that I already fear I knew”*. P5—Parent—genetic cause found
Q14	*“All the stuff I’ve actually found is because I’ve Googled stuff, not because anyone has sat down and gone through like oh maybe you’d find this useful.”* P6—Parent—VUS result
Q15	*“It’s my genome, it’s unique to me, it’s something that defines who I am and it’s part of my legacy going forwards and part of my ancestry, so it’s kind of, you know, the 3bn base pairs that make up my genome are quite important personal data.”* P31—Patient—no genetic cause found
Q16	*“They’ve already said to me that children can get it. Like, she’s my daughter, I’ve got it. There is a possibility that she could get it. It’s always going to be in your mind isn’t it? And it’s quite frightening.”* P14—Patient—no genetic cause found
Q17	*“It could be she’s affected by it so then I’m also wondering does she have it and it kind of fills me with anxiety, like, it scares me a bit to not know”*. P40—Parent—VUS result
Q18	*“He’s such a full of beans child that he reassures me. Then knowing that things can be progressive I, yeah, I just really worry.”* P5—Parent—genetic cause found
Q19	*“I’d like to be a mother, so I am obviously massively thinking about passing this gene on”*. P19—Patient—genetic cause found

### “As soon as you say he has got a diagnosis, they want to help you”: practical and social benefits of GS

#### The value of a label

Many living with no diagnosis described the challenge of explaining the condition to others. For instance, one parent, whose child was overweight, described how a genetic diagnosis would have made it easier to explain to school staff and social services that his increased appetite was related to his condition, and not because he was being overfed (Table 3: Q3). For these participants, the social and practical value of having a genetic diagnosis was evident. One patient described how receiving a diagnosis had now made it easier to explain his neurological condition to his family. Others talked about how having a diagnosis now entitled their child to certain services and financial support (Table 3: Q4). There was, however, little practical or social impact for those who received a no primary finding result—particularly those who did not perceive themselves to be seriously unwell (Table 3: Q5).

#### Behavioural changes for both probands and relatives

Behavioural impacts, such as changes to diet or lifestyle, were reported by some. Some reported the result also led to behavioural changes for other family members. One patient described how her sons “tend to use sunscreen” and “look after themselves a bit better” since learning about her genetic diagnosis for a hereditary condition that predisposes her to skin cancers.

#### Clinical care remained largely unchanged

In a handful of cases, receiving a diagnosis had a direct impact on clinical care; four parents and one patient described how having a diagnosis allowed closer monitoring of family members (Table 3: Q6). Receiving a VUS could also lead to changes, such as being seen by a specialist team or being included in clinical research (Table 3: Q7). However, most reported that clinical care remained “absolutely the same” regardless of whether a genetic cause had been identified (Table 3: Q8). This was particularly so for those who had been living with a clinical diagnosis for many years (such as lipoedema or polycystic kidney disease) and were being medically managed accordingly (Table 3: Q9).

### “It was just nice to have confirmation”: relief in many forms

#### Relief and reassurance

Relief and reassurance after receiving a result were commonly described emotions and were reported more often by those for whom a genetic cause had been found, and more often by parents than patients. For example, one parent who received a diagnosis for her son’s developmental delay, talked about feeling relieved “to know that there is something there”, whilst another described relief that her worries about her son’s eye condition were justified.

#### Relief from guilt and self-blame

A diagnosis could also provide relief from guilt. One patient described how his diagnosis had let him “take any self-blame out of it”. Similarly, one parent described how the diagnosis had alleviated her guilt that lifestyle choices during her pregnancy had caused her child’s condition (Table 3: Q10).

#### Relief when conditions are ruled out

Relief was also experienced in the context of being able to rule out conditions. One parent described relief that a particular genetic variant had not caused her daughter’s kidney condition. For others, it was the lack of a genetic diagnosis that provided reassurance that there was a low risk of passing the condition on (Table 3: Q11).

### Disappointment, uncertainty, frustration, and sadness

#### “I wasn’t expecting miracles, I just wasn’t expecting nothing”: disappointment and sadness when reality does not live up to expectations

In most cases, more negative emotions, like disappointment and sadness, were experienced by those with a no primary finding result, with parents expressing these feelings more often than patients. Often, the source of this disappointment could be attributed to a mismatch between people’s expectations of receiving a diagnosis and their actual results. Whilst some had tried to be “open-minded”, it was clear that many parents had pinned their hopes on finding a genetic cause. One couple, for instance, who had put “hope in the technology” of GS, described grief and sadness at their result not providing details about the heritability of their son’s incredibly rare neurological condition (Table 3: Q12). Notably, sadness was not limited to those who did not receive a diagnosis. One parent described how the reality that there was, in fact, a genetic cause for her child’s rare condition had left her feeling upset (Table 3: Q13).

#### Feeling uninformed left some people frustrated

For a few participants, frustration was evident: one parent described searching for information online because he felt that the health professional had not provided enough details about his VUS result (Table 3: Q14), whilst a patient with a no primary finding result was frustrated at the lack of access to his genetic information (Table 3: Q15).

#### Living with uncertainty: anxiety about the future

Some parents who did not have a genetic diagnosis for their child talked about feeling anxious, even frightened for the future. Concern for the health of other family members was also an issue for several participants who had a no primary finding result (Table 3: Q16). There was also evidence of anxiety when a VUS was found, manifesting for one parent as an “extra kind of layer of vigilance” of their child’s condition. One parent described the uncertainty following a VUS as “quite difficult to live with” and another was anxious about the implications for siblings (Table 3: Q17). Interestingly, participants with a diagnosis could also feel anxiety about an uncertain future that was linked to the prognosis of the diagnosed condition (Table 3: Q18) or the possibility of passing the gene on (Table 3: Q19).

#### Feeling alone despite receiving a diagnosis

Two parents reported feelings of isolation when a very rare disease was diagnosed. One parent felt unsure about where to turn after the diagnosis because the condition was so rare, whilst the other parent felt isolated and worried at the prospect of clinicians having never seen her child’s condition before.

## Discussion

Our mixed methods study examined participants’ experiences of receiving GS results in the 100kGP. We found that DR was low and, in general, there was minimal negative psychological impact of receiving a GS result. An important distinction, however, is that levels of distress and feelings of uncertainty were higher for parents than for patients. In line with the survey results, in which psychological impact was similar for those with and without a diagnosis, the interviews further highlighted that a wide range of individual social, practical, behavioural and emotional impacts are evident regardless of whether a genetic diagnosis is obtained. Similar findings have been noted in other studies exploring the impact of genetic test results on parents of children with rare and undiagnosed diseases [16, 18].

In keeping with other research exploring experiences of GS [17, 28], both our quantitative and qualitative findings indicated that DR was low. Notably, this did not differ across parent and patient groups and was the case whether or not participants had received a genetic diagnosis. The interviews suggest that low DR was frequently linked to altruistic intentions of benefiting research and others. As GS shifts from being offered as part of a research project to being offered more widely in routine clinical care, it will be important to assess whether DR continues to be low, regardless of setting.

Interviews with participants revealed there to be positive psychosocial impacts of GS results. In line with other qualitative studies [28, 29], relief was evident following receipt of a genetic diagnosis, particularly for parents. The power of having a diagnosis for legitimising the condition and bringing about a sense of validation was also seen. For those who had been struggling with the uncertainty of not having a diagnosis, being able to attribute a cause for their or their child’s condition could be helpful practically by facilitating access to specialist equipment and educational and social support. It could also be helpful psychologically and emotionally, providing additional confidence when speaking to both medical professionals and family members. Comparable descriptions of empowerment from obtaining a genetic diagnosis have been described elsewhere [30] and research has supported the value of empowerment as a mechanism not only for driving patients’ healthcare choices [31], but also for coping with the condition on a day-to-day basis [32]. Our findings, therefore, provide further support for the notion that receiving a diagnosis can be instrumental in equipping patients and parents with the tools to play an active role in their own or their child’s healthcare and, where relevant, their social and educational needs.

In terms of the negative emotional impact of receiving a GS result, our survey findings showed them to be minimal and not to differ between those with and without a diagnosis. This is in keeping with a recent meta-analysis [10] and other work showing no significant increase in distress or uncertainty amongst participants who have elected to receive genetic research results [33]. Our interviews, however, clearly show individual variation in experiences and suggest that, for some participants, there were negative experiences associated with GS result disclosure. For those with a no primary finding or a VUS result, frustration and disappointment were described, mirroring findings from other studies exploring the return of GS results [16, 18, 19, 34], and can be viewed as being inextricably linked with the uncertainty of living with an undiagnosed condition [35]. Similar to findings reported by Donohue et al. [19], we also noted that disappointment was greater if initial expectations of receiving a diagnostic result were elevated and that, even in cases where a diagnosis was found, sadness and isolation, linked to the rareness of the condition, were experienced.

Our findings have significant implications. First, they demonstrate the importance of providing patients with realistic expectations during pre-test counselling about the possible implications of GS. Second, they show that assumptions about what support is needed should not be made on the basis of the GS result; receiving a genetic diagnosis does not negate emotions like uncertainty and anxiety and professionals should take this into consideration during post-test conversations. Third, from a methodological perspective, the contrast between the minimal harms identified in the survey (which capture a snapshot of an experience) and the negative emotions expressed in our interviews (which provide scope for deep exploration) highlights the importance of integrating quantitative with qualitative findings for building a comprehensive view of people’s experiences.

Another finding, significant to our research questions was that, whilst our survey results indicated low testing-related distress overall, levels of distress and uncertainty were significantly higher for parents than patients. This was reflected in our interviews where the negative reactions to receiving a GS result were conveyed more viscerally by parents. Such findings could be attributed to variation in perceived quality of life, with patients who have lived with their condition for many years having a different perspective to parents who do not know how the condition will unfold for their child. Another possibility is that parents may invest greater hope in the potential life-changing outcomes of these types of genetic tests and, therefore, experience disheartenment when these expectations are not realised [36, 37]. Taken together, and in line with recent work highlighting the grief-like emotions that parents report after their child’s diagnosis of a rare genetic condition and the important role for emotional support after testing [38], insights from our study make clear the importance of providing care that is adaptable to individual psychological needs.

## Strengths and limitations

Integration of the quantitative and qualitative findings has strengthened the validity of the findings and given a richer understanding of people’s experiences. Furthermore, the research was conducted at multiple hospital sites, increasing the generalisability of the findings. A key limitation, however, was the small proportion of participants who reported receiving a result at the time the survey was administered and respondent bias which may have been towards self-selected participants with strong feelings about GS or those more inclined to engage with the research team. Finally, as this is a cross-sectional study, responses can only give a snapshot experience at a given time.

## Conclusions

With the expanding use of GS in clinical care, achieving a faster final diagnosis for people with rare conditions is frequently highlighted as a key priority [39]. Whilst this is without doubt an important goal, our research highlights that policymakers and healthcare providers also need to put in place appropriate measures that consider the potential variation in the emotional and psychological needs of patients receiving GS results regardless of the type of result. Lessons for policy and practice for delivering GS in the future are timely as genomic medicine matures within mainstream care in the NHS. As such, further research with patients and families undergoing GS in the NHS Genomic Medicine Service will be important.

## Supplementary information

Example of T2 survey and topic guide - Parent version

Characteristics of T2 survey respondents who received a GS result.

Levels of decision regret split by Participant type and Diagnosis status.

Diagnosis status prior to GS and outcome of GS results.

## Data Availability

The datasets generated and analysed during the current study are available from the corresponding author on reasonable request.
