# Supplementary material for: Participant experiences of genome sequencing for rare diseases in the 100,000 Genomes Project: a mixed methods study
Source: Eur J Hum Genet. 2022 Mar 9;30(5):604–10. doi: 10.1038/s41431-022-01065-2 (PMC9091267; doi:10.1038/s41431-022-01065-2)
Supplement: Supplementary file 1 — Example of T2 survey and topic guide - Parent version [file 41431_2022_1065_MOESM1_ESM.pdf]

The 100,000 Genomes Project: Patients' Experiences and Informed Choices

One year follow-up questionnaire

You have been asked to complete this one-year follow-up questionnaire because you are the **parent of a child who has a rare condition**, because you and your child were offered **whole-genome sequencing** as part of the **100,000 Genomes Project**, and because you **previously completed the baseline questionnaire** for this study around one year ago. This questionnaire usually takes about 15 minutes to complete.

Thank you very much for taking the time to complete this second questionnaire. All participants who return this questionnaire completed will receive a **£10 voucher** as a token of appreciation for their time and contribution.

Your answers to these questions are important to us, and will help improve how whole-genome sequencing is delivered in the future. We really appreciate you taking the time to take part in this study.

For office use only:

Section 1: Attitudes

This section is about your thoughts and feelings about having whole-genome sequencing done as part of the 100,000 Genomes Project.

Please indicate whether you agree or disagree with the following statement by ticking the appropriate box:

	Strongly disagree	Disagree	Neither agree nor disagree	Agree	Strongly agree
**1.** I have a clear understanding of what whole-genome sequencing is	☐	☐	☐	☐	☐

For each of the following four questions, please circle the number from 1 to 5 on the scale that best describes how you feel at the moment.

2. For me and my child, having whole-genome sequencing is:

Harmful 1 2 3 4 5 Beneficial

3. For me and my child, having whole-genome sequencing is:

Unimportant 1 2 3 4 5 Important

4. For me and my child, having whole-genome sequencing is:

A bad thing 1 2 3 4 5 A good thing

5. For me and my child, having whole-genome sequencing is:

Not helpful 1 2 3 4 5 Helpful

Please read the statements below about having whole-genome sequencing done as part of the 100,000 Genomes Project. Tick the boxes below to let us know how much you agree or disagree with each statement.

	Strongly disagree	Disagree	Neither agree nor disagree	Agree	Strongly agree
**6.** I feel that taking part could help my child	☐	☐	☐	☐	☐
**7.** I am worried about my personal health information being stored in a large data repository	☐	☐	☐	☐	☐
**8.** I feel that taking part could lead to better medical treatments	☐	☐	☐	☐	☐
**9.** I feel that taking part could help my child get a diagnosis	☐	☐	☐	☐	☐
**10.** I am worried about academic researchers having access to my health information	☐	☐	☐	☐	☐
**11.** I feel that taking part could help other people	☐	☐	☐	☐	☐
**12.** I am worried that my health information might be used by insurance companies	☐	☐	☐	☐	☐
**13.** I feel that taking part could help me personally	☐	☐	☐	☐	☐
**14.** I am worried about drug companies having access to my health information	☐	☐	☐	☐	☐
**15.** I am worried about how I will feel if I learn I have a high risk of developing a serious disease	☐	☐	☐	☐	☐
**16.** I am worried about how the NHS will use my personal health information	☐	☐	☐	☐	☐
**17.** I feel that taking part could advance medical research	☐	☐	☐	☐	☐
**18.** I am worried about companies that make diagnostic tests having access to my health information	☐	☐	☐	☐	☐
**19.** I feel that taking part could identify the underlying cause of my child’s condition	☐	☐	☐	☐	☐

Section 2: Your decision about whole-genome sequencing

When you and your child were invited to take part in the 100,000 Genomes Project, you were asked whether you would like to take part and receive whole-genome sequencing results relating to your child's rare condition. In this section, we are interested in knowing what your feelings are about this.

1. Which of the following options did you choose? Please tick one.

☐ **Option 1:** I chose to take part in the 100,000 Genomes Project and receive any results from whole-genome sequencing relating to my child's rare condition.

☐ **Option 2:** I chose not to take part in the 100,000 Genomes Project.

Please reflect on the decision that you made about taking part in the 100,000 Genomes Project. For each of the following statements, please indicate how strongly you agree or disagree with these statements by ticking the appropriate boxes.

	Strongly disagree	Disagree	Neither agree nor disagree	Agree	Strongly agree
**2.** It was the right decision	☐	☐	☐	☐	☐
**3.** I regret the choice that was made	☐	☐	☐	☐	☐
**4.** I would go for the same choice if I had to do it over again	☐	☐	☐	☐	☐
**5.** The choice did me a lot of harm	☐	☐	☐	☐	☐
**6.** The decision was a wise one	☐	☐	☐	☐	☐

7. Have you received a result from whole-genome sequencing relating to your child's rare condition?

Yes	
No	
Not sure	
Not applicable (chose not to take part)	

If you answered 'Yes' to Question 7 above, please continue on to Question 8 below. If you answered 'No', 'Not sure' or 'Not applicable (chose not to take part)', please skip ahead and go straight to Section 4 on page 7.

8. When did you receive the result relating to your child's rare condition from whole-genome sequencing?

Less than two weeks ago	
Between two weeks and three months ago	
Around 3 to 6 months ago	
Around 6 to 12 months ago	
More than 12 months ago	

9. What was the result relating to your child's rare condition from whole-genome sequencing?

A genetic cause for my child's rare condition was found	
A genetic cause for my child's rare condition was not found	
Not sure	
Other	

Section 3: Main results from whole-genome sequencing relating to your child's condition

The following questions are about some specific responses you may have had after receiving your child's main result from whole-genome sequencing relating to their condition. Please indicate whether you have experienced each statement never, rarely, sometimes, or often, in the past week.

	Never	Rarely	Sometimes	Often	Not applicable
1. Feeling upset about your child's result	☐	☐	☐	☐	☐
2. Feeling sad about your child's result	☐	☐	☐	☐	☐
3. Feeling anxious or nervous about your child's result	☐	☐	☐	☐	☐
4. Feeling guilty about your child's result	☐	☐	☐	☐	☐
5. Feeling relieved about your child's result	☐	☐	☐	☐	☐
6. Feeling happy about your child's result	☐	☐	☐	☐	☐
7. Feeling a loss of control because of your child's result	☐	☐	☐	☐	☐
8. Having problems enjoying life because of your child's result	☐	☐	☐	☐	☐
9. Worrying about your child's health because of their result	☐	☐	☐	☐	☐
10. Being uncertain about what your child's result means for their health	☐	☐	☐	☐	☐
11. Being uncertain about what your child's result means for your family's health	☐	☐	☐	☐	☐
12. Having difficulty making decisions about your child having surgery or medical tests because of their result	☐	☐	☐	☐	☐
13. Being uncertain about what the result means for the management of my child's rare condition	☐	☐	☐	☐	☐
14. Feeling frustrated that there are no definite guidelines for your child based on their result	☐	☐	☐	☐	☐
15. Thinking about your child's result has negatively affected your work or family life	☐	☐	☐	☐	☐
16. Feeling concerned that your child's result will affect your family's insurance	☐	☐	☐	☐	☐
17. Feeling regret about getting your child's result	☐	☐	☐	☐	☐

Please indicate whether you have ever experienced the following things since receiving your child's result.

	Yes	No	Not sure
18. Have you had any difficulty talking about your child's result with family members?	☐	☐	☐
19. Have you felt that your family has been supportive during the whole-genome sequencing process?	☐	☐	☐
20. Have you been satisfied with family communication about your child's result?	☐	☐	☐
21. Have you worried about the whole-genome sequencing process causing conflict within your family?	☐	☐	☐

Receiving a result from whole-genome sequencing regarding my child's rare condition has…	Yes	Not yet / too soon to say	Not sure / not applicable	No
1. Led to improvements in my child's treatment and/or care plan	☐	☐	☐	☐
2. Led to improvements in my child's access to physical health care	☐	☐	☐	☐
3. Led to changes in my child's lifestyle	☐	☐	☐	☐
4. Improved my child's access to support from social services	☐	☐	☐	☐
5. Meant that we have been able to connect with specific rare disease support groups, e.g. Facebook or in-person groups	☐	☐	☐	☐
6. Improved my child's self-confidence	☐	☐	☐	☐
7. Improved our communication with medical professionals	☐	☐	☐	☐
8. Given us useful information for life planning	☐	☐	☐	☐
9. Given us useful information for reproductive decision-making	☐	☐	☐	☐
10. Other impact or outcomes	☐	☐	☐	☐

If 'Other impact or outcomes', please specify:

We are interested to hear more about the impact that your child's result from whole-genome sequencing has had on you and your family. Please could you provide further details about any effects on by writing these in the box below.

Section 4: Your decision about ‘additional findings’ relating to your health

When you and your child were invited to take part in the 100,000 Genomes Project, you were asked whether you would like the project scientists to look for ‘additional findings’ relating to your health (e.g. cancer predisposition). In this section, we are interested in knowing what your feelings are about this.

1. Which of the following options did you choose? Please tick one.

☐ **Option 1:** I chose for the project scientists to look for ‘additional findings’ relating to my health.

☐ **Option 2:** I chose for the project scientists not to look for ‘additional findings’ relating to my health.

☐ **Option 3:** I can’t remember what I chose for the ‘additional findings’ relating to my health.

Please reflect on the decision that you made about receiving ‘additional findings’ relating to your health. For each of the following statements, please indicate how strongly you agree or disagree with these statements by ticking the appropriate boxes.

	Strongly disagree	Disagree	Neither agree nor disagree	Agree	Strongly agree
**2.** It was the right decision	☐	☐	☐	☐	☐
**3.** I regret the choice that was made	☐	☐	☐	☐	☐
**4.** I would go for the same choice if I had to do it over again	☐	☐	☐	☐	☐
**5.** The choice did me a lot of harm	☐	☐	☐	☐	☐
**6.** The decision was a wise one	☐	☐	☐	☐	☐

7. Have you received any ‘additional findings’ relating to your health from whole-genome sequencing?

Yes	
No	
Not sure	

If you answered ‘Yes’ to Question 7 above, please continue on to Question 8 below. If you answered ‘No’, ‘Not sure’ or ‘Not applicable (chose not to take part)’, please skip ahead and go straight to Section 6 on page 9.

8. When did you receive the ‘additional finding’ relating to your health from whole-genome sequencing?

Less than two weeks ago	
Between two weeks and three months ago	
Around 3 to 6 months ago	
Around 6 to 12 months ago	
More than 12 months ago	
Not applicable (have not received any ‘additional findings’)	

Section 5: 'Additional findings' relating to your health

The following questions are about some specific responses you may have had after receiving your additional findings relating to your own health from whole-genome sequencing. Please indicate whether you have experienced each statement never, rarely, sometimes, or often, in the past week.

	Never	Rarely	Sometimes	Often	Not applicable
1. Feeling upset about your 'additional finding'	☐	☐	☐	☐	☐
2. Feeling sad about your 'additional finding'	☐	☐	☐	☐	☐
3. Feeling anxious or nervous about your 'additional finding'	☐	☐	☐	☐	☐
4. Feeling guilty about your 'additional finding'	☐	☐	☐	☐	☐
5. Feeling relieved about your 'additional finding'	☐	☐	☐	☐	☐
6. Feeling happy about your 'additional finding'	☐	☐	☐	☐	☐
7. Feeling a loss of control because of your 'additional finding'	☐	☐	☐	☐	☐
8. Having problems enjoying life because of your 'additional finding'	☐	☐	☐	☐	☐
9. Worrying about your health because of your 'additional finding'	☐	☐	☐	☐	☐
10. Being uncertain about what your additional 'finding means for your health'	☐	☐	☐	☐	☐
11. Being uncertain about what your 'additional finding' means for your family's health	☐	☐	☐	☐	☐
12. Having difficulty making decisions about having surgery or medical tests because of your 'additional finding'	☐	☐	☐	☐	☐
13. Being uncertain about what the 'additional finding' means for the management of your health	☐	☐	☐	☐	☐
14. Feeling frustrated that there are no definite guidelines for you based on your 'additional finding'	☐	☐	☐	☐	☐
15. Thinking about your 'additional finding' has negatively affected your work or family life	☐	☐	☐	☐	☐
16. Feeling concerned that your 'additional finding' will affect your insurance	☐	☐	☐	☐	☐
17. Feeling regret about getting your 'additional finding'	☐	☐	☐	☐	

Please indicate whether you have ever experienced the following things since receiving your 'additional finding'.

	Yes	No	Not sure
18. Have you had any difficulty talking about your 'additional finding' with family members?	☐	☐	☐
19. Have you felt that your family has been supportive during the 'additional findings' process?	☐	☐	☐
20. Have you been satisfied with family communication about your 'additional finding'?	☐	☐	☐
21. Have you worried about the 'additional findings' process causing conflict within your family?	☐	☐	☐

Section 6: Knowledge about whole-genome sequencing

In this final section, we would like to ask you some questions that will help us develop a new measure of understanding of whole-genome sequencing.

For each of the following statements, please indicate whether you think each statement is “True” or “False” by ticking the appropriate box. If you don’t know or are not sure, this is absolutely fine -- please just tick the “Don’t know” box.

	True	False	Don’t know
**1.** A person’s genome is their body’s ‘instruction manual’ containing the information needed to make them, run them and repair them	☐	☐	☐
**2.** Scientists know what all parts of the genome do	☐	☐	☐
**3.** There are uncertainties about what a person’s genome can tell them	☐	☐	☐
**4.** Whole-genome sequencing may not provide a person with any meaningful information about their health	☐	☐	☐
**5.** A person’s genome is the complete set of cells in their body	☐	☐	☐
**6.** Whole-genome sequencing involves looking at around half of the DNA in a genome	☐	☐	☐
**7.** A person’s genome is the 1% of their DNA that makes proteins	☐	☐	☐
**8.** The effects of all DNA variants identified through whole-genome sequencing on disease are known	☐	☐	☐
**9.** Whole-genome sequencing is different to other genetic tests because it looks at almost all of a person’s DNA, rather than only a small bit of it	☐	☐	☐

Section 6. Final Comments

We are very interested in any additional thoughts, concerns or comments you might have regarding the process of whole-genome sequencing and taking part in the 100,000 Genomes Project so far, so that we can improve the process for other people going forward. We are interested in your thoughts regardless of whether or not you have received any results. Do you have any further suggestions for things you would like done differently, or things you thought were good and worked well? Please write any further comments you have in the box below.

Thank you again for taking the time to complete this questionnaire. Your answers to these questions are important to us, and will help improve how whole-genome sequencing is delivered in the future. We really appreciate you taking the time to take part in this study.

You can find further information about the 100,000 Genomes Project here: www.genomicsengland.co.uk

Topic guide: In-depth interviews with patients/parents in receipt of a GS result

1. Motivations and expectations

1. First, would you tell me a bit about your condition and impact it has on you/your child's life?
- o What has it been like for you/your child, not having a diagnosis/genetic cause for condition? [Prompt: how did it impact you/your child's clinical care/treatment, everyday life e.g. schooling, work, access to benefits, housing etc]
- o What are the pathways you have been down to find a diagnosis?

2. What were you hoping to get from taking part in the 100,000 Genomes Project?
- o learning about a diagnosis
- o potential treatment
- o contribute to research

3. What were your expectations in terms of whether you would get a diagnosis?
- o Do you recall what the health professional consenting you into the project said about whether you would get a diagnosis?

4. What was it like for you waiting for your results?

2. Experience of receiving results including a negative result

You've since received results from the 100,000 Genomes project, I'd like to talk a little bit about what it was like getting that result.

5. Could you tell me what result you received?
- o Was there a diagnosis or clear genetic cause for the condition?
- o Does it provide answers? What questions does it answer for you? [Prompt: Whether it was inherited or de novo, prognosis, a reason why it happened]
- o Was it the result you were expecting? Was there anything unexpected?

6. How long did it take for you to receive your/your child's results?
- o Longer/shorter than expected?
- o Was that an acceptable amount of time for you?

7. When you were given your result, how were you informed about it? [Letter, phone call]
- o Who delivered the results?
- o Was an appointment made to discuss results?

8. What would you say about the way your result was communicated to you?
- o Was the explanation of your result easy to understand?
- o Was there any confusion e.g. was the terminology difficult to understand?
- o Was there a discussion around what the result meant in terms of you/your child's care?
- o Do you think the way that you were given results was appropriate?
- o Could anything have been done differently or better?

- Was the discussion handled sensitively?
- How long was the appointment?
- Were you given anything in writing?

9. Have you been given advice about next steps?
- Do you know whether your results have been shared with other health professionals involved in you/your child's care?
- If yes, was this done effectively?
- Have you been signposted to any support groups following the result?

3. Impact of receiving a diagnosis / identifying genetic cause

I'd like to talk now about what the impact has been for you/your child getting a result.

10. Has there been a clinical impact from receiving a diagnosis/finding a genetic cause for the condition?
- What has the result meant in terms of managing you/your child's condition e.g. further medical tests? Change in medication? Change in healthcare team?
- Any other impact in terms of the way you manage you/your child health? e.g healthy eating, exercise?
- Have you been given any *new* information about your child's condition e.g. related to prognosis?

11. What has been the emotional impact of receiving a diagnosis? E.g. closure, end to diagnostic odyssey, relief?

12. What has been the practical or social impact of receiving a diagnosis?
- In terms of social services/schooling/work/benefits
- Have you come across any issues in terms of lack of understanding or recognition of the disorder e.g. with other non-clinical professionals?
- And if so has this been a barrier to ongoing practical support or benefits?
- Has there been an impact on your relationship with family or friends now that you have a diagnosis?
- Do you think it has an impact in terms of any other important relationships in your life?
- Has it had an impact in terms of having other children?

13. What do you think have been the main positive consequences from having a result?

14. Have there been any negative consequences from having a result?
- Any negative clinical consequences e.g. prognosis more serious than anticipated, change to a new clinical team?
- Any negative emotional outcomes e.g. anxiety, fear, guilt, disappointment, frustration
- Any negative social consequences e.g. loss of support from 'undiagnosed' community

4. Impact on receiving a result that did <u>not</u> provide a clear diagnosis/genetic cause e.g. “nothing found so far”

I’d like to talk now about what it has been like getting a result that did not provide you/your child with a diagnosis.

15. Has this result had any <u>clinical</u> impact in terms of you/your child’s care? Prompt: Has it ruled any conditions out that you previously suspected? Further testing? Change in medication? Change in healthcare team? has been the impact of the result on you/your child’s care?

16. What has been the <u>emotional</u> impact of not receiving a diagnostic result? E.g. disappointment, frustration, relief?

17. Has there been any <u>practical or social</u> impact of not receiving a diagnostic result?

18. Have there been any <u>negative consequences</u> from not having a diagnostic result?

19. Have there been any <u>positive consequences</u> from not having a diagnostic result?

20. What are your thoughts now about whether you will get a diagnosis?

21. What has been the guidance from the clinicians in terms of next steps?

22. Has there been any discussion about results being reinterpreting at a later date?

5. Additional findings

23. Do you remember whether you opted to receive additional findings?

24. Have you received any results about the AFs? (likely to be no!)

25. Have you had any further thoughts about your decision to receive/not receive AFs since the appointment? [Prompt: regret, anxious, not thought about it]

6. Reflections on taking part

26. Reflecting back on the process since receiving the result, how do you feel about taking part in the 100,000 Genomes project?
- Have you got the answers that they wanted?
- Has it helped you in anyway?

27. Do you have any regrets about taking part?
- If so, could you explain what those are?
- What would you have done differently?

28. Do you have any other thoughts or comments you would like to share?

References used to develop questions:

Li et al. (2018): *Caregivers' perception of and experience with variants of uncertain significance from whole exome sequencing for children with undiagnosed conditions*

Wynn et al. (2017): *Diagnostic exome sequencing in children: A survey of parental understanding, experience and psychological impact*

Krabbenborg et al. (2016): *Understanding psychosocial effect of WES test results on parents of children with rare disease.*

Sapp et al. (2013): *Parental attitudes, values, and beliefs towards the return of results from exome sequencing in children.*

Chassagne et al. (2019) *Exome sequencing in clinical settings: preferences and experiences of parents of children with rare diseases (SEQUAPRE study)*
